# When should a rare inherited connective tissue disorder be suspected in bicuspid aortic valve by primary-care internists and cardiologists? Proposal of a score

**DOI:** 10.1007/s11739-020-02458-1

**Published:** 2020-09-19

**Authors:** Guglielmina Pepe, Betti Giusti, Stefania Colonna, Maria Pia Fugazzaro, Elena Sticchi, Rosina De Cario, Ada Kura, Elisa Pratelli, Daniela Melchiorre, Stefano Nistri

**Affiliations:** 1 Marfan Syndrome and Related Disorders Regional Referral Center, Careggi Hospital, Viale Gaetano Pieraccini, 50139 Florence, Italy; 2 Research and Innovation Center for Marfan Syndrome and Related Disorders, Careggi Hospital, Florence, Italy; 3 Department of Experimental and Clinical Medicine, Section of Critical Medical Care and Medical Specialities, University of Florence, Florence, Italy; 4 Atherothrombotic Diseases Tuscany Referral Center, Careggi Hospital, Florence, Italy; 5 Outpatient Cardiology Unit, Health District 1 ULSS 6, Vigonza and Carmignano di Brenta, Padua, Italy; 6 Cardiology Service, CMSR Veneto Medica, Altavilla Vicentina, Italy; 7 Specialization in Physical and Rehabilitation Medicine, Recovery and Rehabilitation Agency, Careggi Hospital, Florence, Italy; 8 Department of Experimental and Clinical Medicine, Section of Rheumatology, University of Florence, Florence, Italy

**Keywords:** Aortopathy, Congenital heart disease, Connective tissue disorders, Bicuspid aortic valve, Marfan syndrome, Primary care

## Abstract

Size threshold for aortic surgery in bicuspid aortic valve (BAV) is debated. Connective tissue disorders (CTDs) are claimed as a clinical turning point, suggesting early surgery in BAV patients with CTD. Thus, we aimed at developing a score to detect high risk of carrying CTDs in consecutive BAVs from primary care. Ninety-eight BAVs without ectopia lentis or personal/family history of aortic dissection were studied at the Marfan syndrome Tuscany Referral Center. Findings were compared with those detected in 84 Marfan patients matched for sex and age. We selected traits with high statistical difference between MFS and BAV easily obtainable by cardiologists and primary-care internists: mitral valve prolapse, myopia ≥ 3DO, pectus carenatum, pes planus, wrist and thumb signs, and difference between aortic size at root and ascending aorta ≥ 4 mm. Clustering of ≥ 3 of these manifestations were more frequent in Marfan patients than in BAVs (71.4% vs 6.1%, *p* < 0.0001) resulting into an Odds Ratio to be affected by MFS of 38.3 (95% confidence intervals 14.8–99.3, *p* < 0.0001). We propose a score assembling simple clinical and echocardiographic variables resulting in an appropriate referral pattern of BAVs from a primary-care setting to a tertiary center to evaluate the presence of a potential, major CTD.

## Introduction

Bicuspid aortic valve (BAV) is the most common congenital heart disease with a prevalence of 1–2% [1–3]. As such, BAV may be commonly evaluated by general practitioner in primary care, as well as internist in multiple settings. It is frequently associated to thoracic aortic aneurysm (TAA) [4–6]. Early studies on BAV reported structural abnormalities of thoracic aortic tissue resembling those detected in Marfan syndrome (MFS) [7]. More recently, a higher percentage of BAV has been reported in Marfan (MF) patients with respect to general population [8, 9], suggesting common pathogenetic mechanisms at least in some patients [3, 9]. Moreover, an absolute increased risk of aortic dissection (AD) has been reported in BAV patients [10, 11]. These issues lead to aggressive recommendations for elective aortic surgery for BAVs similar to those adopted in MF patients. Subsequently, however, long-term independent studies [12–14] produced evidences supporting new guidelines providing separate threshold for aortic surgery in MF and BAV patients [12–14]. Indeed, a recent meta-analysis [15] indicates a low risk of rupture or dissection of moderately dilated aortas without evidence that those related to BAV perform more poorly than others. These data further support the recommended threshold of 55 mm for intervention in BAVs without risk factors [12–14]. Nonetheless, there is still dispute on this topic with some suggesting more aggressive surgical approach in BAV patients and others expanding the risk factors to specific phenotypes (i.e. the root phenotype [5, 16].

Reported clinical and genetic overlaps between MFS and BAV [3, 9, 17] suggesting that early detection of syndromic connective tissue disorders (CTDs), underlying aortic ectasia in BAVs, might be useful to address patients to distinct decision-making algorithms towards aortic surgery [17]. However, direct referring of each BAV patient with TAA for a clinico-genetic assessment looks largely unpractical and unsustainable, since BAV is prevalent and commonly associated to significant aortic dilatation.

Thus, we aimed at searching for an easily applicable clinical score supporting clinical cardiologists, general practitioners and internists to detect those BAVs with a high risk of significant CTDs, prompting their appropriate referral to a tertiary center for clinico-genetic assessment.

## Materials and methods

### Patients

Ninety-eight patients with BAV without ectopia lentis or personal/family history of AD and CTDs (i.e. Marfan syndrome, Loeys-Dietz syndromes, vascular Ehlers-Danlos) were consecutively referred by cardiologists of primary-care facilities to the MF Tuscany Referral Center for being evaluated by one clinical geneticist to investigate the presence of MFS or other syndromic CTDs [18]. According to Ghent2 criteria for the diagnosis of Marfan syndrome [19], most of patients with BAV presented neither two clinical signs nor a clinical and a genetic one. The 60/98 BAV patients which accepted to undergo FBN1 mutation analysis turned out do not carry any FBN1 mutation.

Eighty-four MF patients, diagnosed by new Ghent criteria [18, 19], similar for sex and age, were extracted from the clinical database of the Center for comparison.

### Transthoracic echocardiography

Aortic size was assessed by 2D transthoracic echocardiography by leading edge-to-leading edge method in parasternal long-axis views, at end-diastole [8, 20]. Bicuspid valves are classified as type 1 (right–left coronary cusp fusion), type 2 (right-non-coronary cusp fusion), and type 3 (left-non-coronary cusp fusion). To take into account phenotypic differences of aortic dilatation in BAV and MF patients [6, 20], we analyzed the difference between aortic size at Valsalva sinuses and at proximal ascending aorta (aortic root (AR)—ascending aorta (AA) diameters = ΔAR-AA) and we categorized patients on the basis of a threshold value of 4 mm derived from 98 control subjects without family history of either BAV or MFS comparable for age and sex.

### Systemic features of Marfan syndrome

Systemic features (SF) collected and reported in Table 1 were assessed by the Senior investigator (GP).

**Table1:** Demographic and clinical characteristics of the studied BAV and MFS patients

FBN1	BAV *N* = 98	MFS *N* = 84	*P* value BAV vs MFS
Age, years	42 (18–72)	43 (18–69)	0.109
Sex Male, *n* (%)	70 (71.4)	65 (77.4)	0.360
Height, cm	176 (150–194)	183 (149–205)	< 0.0001
Weight, kg	74 (46–112)	75 (33–115)	0.540
Aortic root diameter, mm	37.5 (26–53)	42.0 (29–56)	< 0.0001
Ascending aortic diameter, mm	38.0 (22–53)	32.0 (24–60)	0.002
ΔAR-AA, mm (IQR)	–0.6 (–5.9–3.0)	9.0 (6.0–12.0)	< 0.0001
**ΔAR-AA > 5 mm**, ***n (%)***	19 (19.4)	77 (91.7)	< 0.0001
Systemic features (SF)			
Mitral valve prolapse* (MVP), *n* (%)	28 (28.6)	62 (73.8)	< 0.0001
Dolicocephaly: face and/or neck, *n* (%)	72 (73.4)	48 (57.1)	0.020
Jaw ipo and/or retrognathic, *n* (%)	76 (77.6)	40 (47.6)	< 0.0001
Pectus carinatum deformity*, *n* (%)	20 (20.4)	36 (42.9)	0.001
Pectus excavatum, *n* (%)	31 (31.6)	35 (41.7)	0.160
Kyphosis, *n* (%)	30 (30.6)	18 (21.4)	0.161
Scoliosis > 20°, *n* (%)	10 (10.2)	41 (48.8)	< 0.0001
Reduced elbow extension, *n* (%)	17 (17.3)	30 (35.7)	0.005
**Wrist and thumb sign**†, *n* (%)	3 (3.1)	23 (27.4)	< 0.0001
**Plain pes planus**, *n* (%)	12 (12.2)	41 (48.8)	< 0.0001
Hindfoot deformities *n* (%)	33 (33.6)	31 (36.9)	0.649
**Myopia > 3 diopters**, *n* (%)	14 (14.3)	32 (38.1)	0.0002
Pneumothorax, *n* (%)	3 (3.1)	7 (8.3)	0.119
Striae, *n* (%)	82 (83.7)	69 (82.1)	0.789

*BAV* bicuspid aortic valve; patients were all assessed according to Ghent-2 criteria (19), *MFS* Marfan syndrome, *ΔAR-AA* Delta Aortic Root—Ascending Aorta diameter, * of any kind, † thumb sign is positive when the entire nail of the thumb projects beyond the ulnar border of the hand which is clenched without any assistance, the wrist sign is positive when the thumb overlaps the terminal phalanx of the fifth digit when it grasps the contra-lateral wrist. **FBN1 gene analysis was performed in 60/98 BAV patients, all the ones which accepted to undergo mutation analysis. All patients (100%) turned out to be negative for FBN1 mutations. Among the other 38 patients, 19 had an aorta diameter with z-score < 2, 3 did not have aortic root ectasia, the remaining 16 had a systemic features’ score between 0 and 3 (11 patients), 4 and 5 (the remaining 5). Furthermore, these last patients did not have mitral valve prolapse or marphanoid aspect. Family history was negative for MFS (data not shown). The results were expressed as median and range or interquartile range (IQR) for continuous variables or percentages for categorical variables. Medians were compared by Mann–Whitney test and categorical variables were analysed by the chi-square test by SPSS package v19 (SPSS Inc., Chicago, IL, USA)

### Informed written consent

All patients gave informed written consent to participate in the study approved by the ethic committee.

### Statistical analysis

The results were expressed as median and range or interquartile range (IQR) for continuous variables or percentages for categorical variables. Medians were compared by Mann–Whitney test, and categorical variables were analysed by the chi-square test using SPSS package v19 (SPSS Inc., Chicago, IL, USA). Statistical significance was accepted at *p* value < 0.05.

## Results

### Demographic, echocardiographic and clinical characteristics of MF and BAV patients

**Fig. 1:**
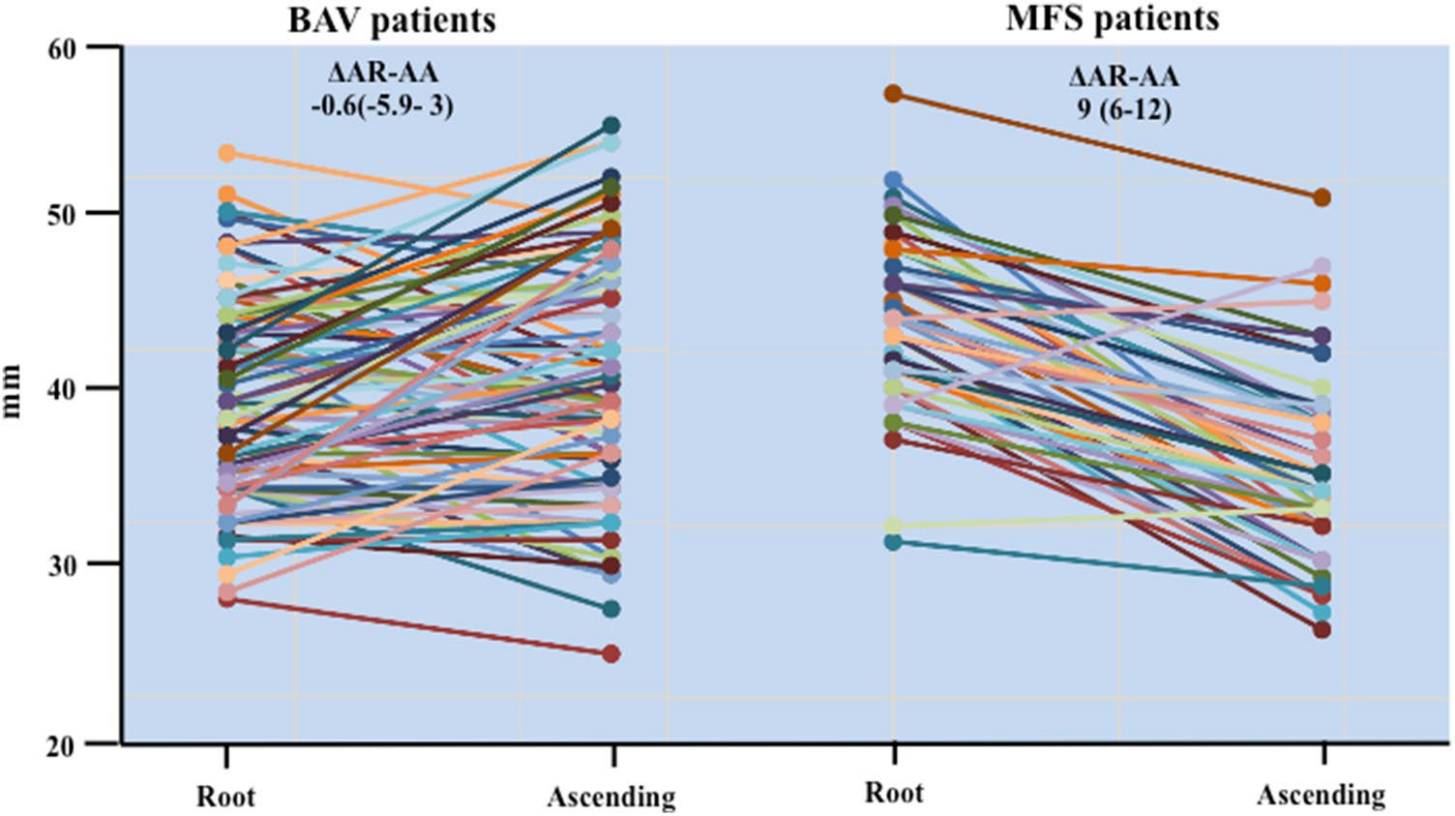
Individual aortic Size at Aortic Root and Ascending Aorta in BAV (left) and MFS (right) patients. BAV patients display a larger ascending aorta diameter respect to root diameter, while the root diameter appears mostly larger in Marfan patients which have also larger aortic size. Aorta in BAV displays a larger ascending vs root aorta diameter. Root diameter and aortic size are larger in MF patients. These data result in a significantly higher ΔAR-AA in MF patients respect to BAV patients (*p* < 0.0001)

Demographic, echocardiographic and clinical characteristics of MF and BAV patients are reported in Table 1. BAV patients had type 1 morphology in 77/98 (78.6%), type 2 morphology in 20/98 (20.4%) and type 3 in 1/98 (1.0%) and were associated to moderate or severe aortic regurgitation in 29/98 patients (29.6%), moderate or severe aortic stenosis in 7/98 patients (7.1%), and moderate or severe calcification in 6/98 patients (6.1%). MF patients were taller, had larger aortic size at aortic root, while smaller ascending aorta, resulting in a significantly higher ΔAR-AA 9.0 (6.0–12.0) vs − 0.6 (− 5.9 to 3.0) mm, respectively, *p* < 0.0001 (Fig. 1).

Indeed, only 19.4% of BAV patients had ΔAR-AA exceeding the value of 4 mm, while 91.7% of MFs satisfied this criterion (Table 1). Among all clinical traits, we selected those with high statistical difference between MFS and BAV (in bold in Table 1) and readily obtainable by cardiologists in a non-referral setting, to develop a score to detect BAVs at high risk of carrying CTDs: mitral valve prolapse, myopia ≥ 3DO, pectus carinatum, pes planus, wrist and thumb signs, and ΔAR-AA ≥ 4 mm.

Patients with 3 or more of these manifestations were *n* = 60 (71.4%) among MFs and *n* = 6 (6.1%) among BAVs (*p* < 0.0001). These 6 BAV patients were similar regarding age, sex and prevalence of moderate-to-severe aortic regurgitation and stenosis to BAVs with < 3 manifestations (data not shown), had smaller aortic size at the aortic root [32.0 (28.0–40.0) mm, vs. 38.3 (25.6–53.0) mm, respectively, *p* = 0.018] and at proximal ascending aorta [31.0 (25.0–37.0) mm vs. 38.5 (22.0–53.0) mm, respectively, p = 0.006]. ΔAR-AA was comparable between BAV patients with ≥ 3 [2.0 (− 2.1 to 4.7) mm] to the remaining BAVs [− 1.0 (− 6.0 to 3.0) mm, *p* = 0.257]; moreover, prevalence of BAV patients with ΔAR-AA > 4 mm was not different between the two groups [*n* = 2 (33.3%) vs *n* = 17 (18.5%), respectively, *p* = 0.373]. At logistic regression analysis, subjects with ≥ 3 of the six manifestations showed an Odds Ratio to be affected by MFS of 38.3 (95% confidence intervals 14.8–99.3), *p* < 0.0001.

## Discussion

The issue of size threshold for aortic surgery in BAV is still debated [22, 23], though more recent guidelines lean towards a more conservative approach in BAV patients with isolated TAA [12–14]. Indeed, an important agreement has been recently reached to indicate surgical repair when the aortic diameter is ≥ 5.5 cm in BAV patients without risk factor (i.e.: family history of aortic dissection or rapid increase in aortic size) or elastopathy, while a lower threshold was maintained for patients with Marfan syndrome”.

The presence of CTD has been suggested as a clinical turning point in this scenario, supporting more conservative attitude in BAV patients without CTD [17]. However, the idea of referring each and every BAV patient with TAA [24] to a tertiary Center is clearly non-realistic due to the prevalence of BAV itself and of BAV-related aortic dilatation [10]. Thus, we studied outpatients with BAV without ectopia lentis or family history of aortic dissection and CTDs (i.e.: clinical conditions supporting per se the need of a clinic-genetic evaluation), consecutively detected in primary care, aiming at identifying signs whose presence strongly suggests the prevalence of CTD. We demonstrate that the combination of three or more characteristics (among those included in our score) (Table 2) identify a very limited number of BAV individuals at real potential risk of CTD, thus deserving an appropriate referral to a tertiary Center specialized in CTDs. This score is specific for Marfan patients but it is not designed to exclude LDS or vEDS patients.

**Table 2:** Scoring system for detecting Marfan syndrome among BAV patients

Score	No	Yes
Mitral valve prolapse		
Myopia ≥ 3DO		
Pectus carinatum		
Pes planus		
Wrist and thumb signs		
ΔAR-AA ≥ 4 mm		
Total score*		

The opportunity to refer the patient at a tertiary Center for MFS evaluation should be considered with a score ≥ 3

^*^Each feature corresponds to a score of 1. Patients with a total score ≥ 3 have an Odds Ratio to be affected by MFS of 38.3 (95% CI 14.8–99.3)

The herein proposed score is based on the combination of systemic traits (myopia > 3 dioptres, pectus carenatum, pes planus, wrist and thumb sign) easily detectable by clinical cardiologists and primary-care internists working in non-referral facility, and echocardiographic characteristics (mitral valve prolapse and ΔAR-AA). This score provides a tool to avoid an inappropriate, systematic referral of all BAVs with TAA to a tertiary center for a clinic-genetic assessment with consequent significant reduction of healthcare costs and patients’ discomfort.

Von Kodolitsch et al. set up a pre-test probability score of Marfan syndrome in a study population characterized by a high rate of positive family history and aortic complications requiring surgery (25). Although our findings display some overlapping features, our score cannot be compared to the previous score since the goal of our work required the choice of different manifestations. In fact, our aim prompted us to select features both with highest differences between BAV and MF patients and easily appliable by internists or clinical cardiologists, usually dealing with the majority of BAVs in a non-referral setting.

**Fig. 2:**
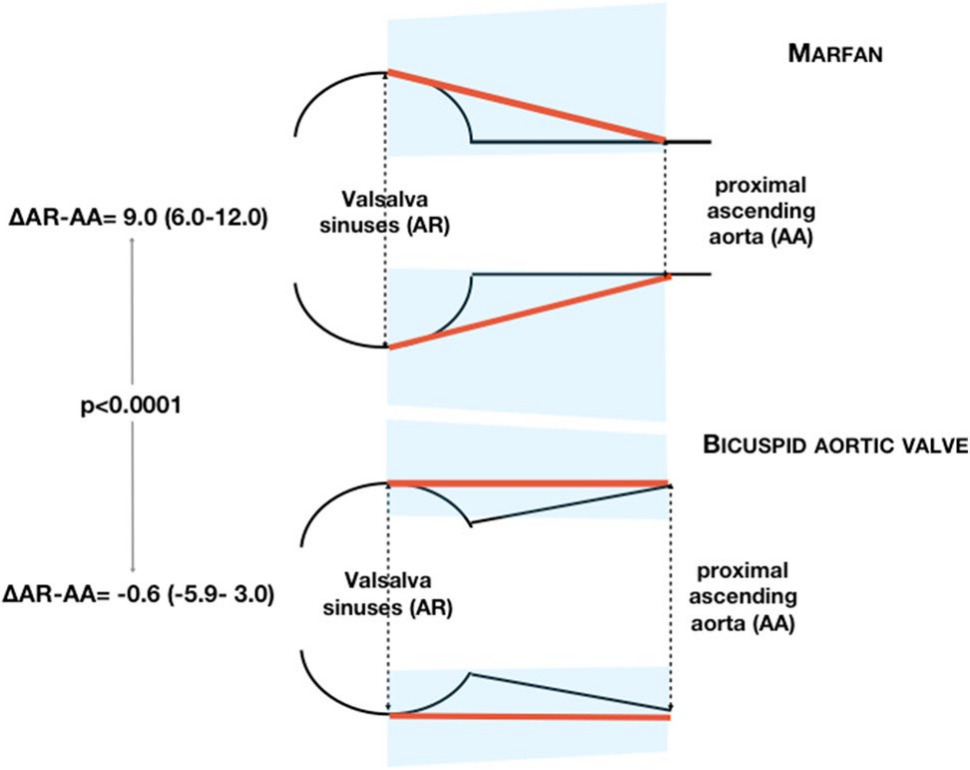
Schematic representation of the echocardiographic evaluation of the aortic root for patients with either Marfan syndrome or bicuspid aortic valve. Aortic size was measured at Valsalva sinuses (AR) and proximal ascending aorta (AA) (dotted, double headed, arrows). Median aortic diameter at each site are linked by a red straight line, with shaded areas addressing interquartile ranges. ΔAR-AA: difference of the aortic size at Valsalva sinuses and at proximal ascending aorta

There are evidences that the so-called root phenotype [26] might represent a more severe form of aortopathy, as recently reviewed [6]. The root phenotype has been found associated with acute aortic events after isolated aortic valve replacement as well as with potentially aortopathy-related genetic variants [9, 27, 28]. Consistently, the root phenotype has been recently included among the adjunctive risk factors to consider when indicating earlier elective surgery for BAV aortopathy [29]. Considering this background, we introduced ΔAR-AA, (Fig. 2), trying to resume the different patterns of aortic dilatation observed in BAV and MF patients [6, 20]. This is consistent with the notion that dilatation of the aortic root is less prevalent in BAV, while it is a recognized pattern in MFS, suggesting different anatomic sites of aortic vulnerability [20]. Noteworthy, the six BAV patients with three or more characteristics suggesting significant CTD, were not distinguishable from the other BAVs based on either aortic valve hemodynamic, aortic size or aortic phenotype (assessed as ΔAR-AA, as a continuous variable or dichotomized at 4 mm). The clinical implication of this finding is that CTD cannot be either suspected or ruled out simply based on BAV-related aortic phenotypes. Apparent discrepancies between our findings and those supporting the peculiarities of the root-phenotype [22, 26, 27] should be seen cautiously, considering differences of clinical settings possibly resulting into relevant differences among study populations. Our study group was detected in a primary-care setting, while the other studies [22, 26, 27] were performed in patients referred to cardiac surgery centers for either evaluation or intervention Thus, we believe that this apparent mismatch should be interpreted as an expression of the wide spectrum of BAV syndrome [9, 10, 30, 31]. This is of particular importance in this moment in which several new parameters of risk stratification have been proposed (i.e.: aortic shape, valve morphotypes and aortic phenotype, fluid-dynamics-related risk markers, circulating biomarkers) whose connection with the risk of acute complications in BAV patients, however, has not been definitely demonstrated [6].

We acknowledge that this study has multiple limitations. We do not have a validation cohort, or we have performed a clinical follow-up of these patients. However, it has been clearly demonstrated that very long follow-up periods in large unselected populations are needed to detect a significant number of aortic events, including surgery and dissection [12, 29]. Thus, we cannot infer that a low estimated score in BAVs without other risk factors [12–14] might favorably influence the shared decision-making process for delayed thoracic aortic intervention (i.e.: at ≥ 55 mm), though we show that it can affect the decision not to refer a patient to a tertiary CTD Center. Prognostic implications of our proposed score, should be of course assessed in larger study groups with appropriate follow-up. However, considering aims of the present observational study, we do not feel that these limitations flaw our findings.

In conclusion, we demonstrate that a simple, though accurate clinical and echocardiographic evaluation, when assembled in a score, results in more appropriate referral pattern of BAV outpatients studied in a primary-care setting to tertiary CTD centers. This finding suggests that internists and clinical cardiologists should further refine their clinical skills in evaluating BAVs, adding to a proper family and personal history taking [12–14], the search of systemic traits and peculiar echocardiographic findings which, when present in combination, strongly support the need of a clinic–genetic evaluation for a potential, major CTD.
